# Investigation on the Potential Use of Polypropylene Mesh for the Reinforcement of Heat-Polymerized PMMA Denture Base Resin

**DOI:** 10.3390/polym14163300

**Published:** 2022-08-12

**Authors:** Kaan Yerliyurt, Sinan Eğri

**Affiliations:** 1 Department of Prosthodontics, Faculty of Dentistry, Tokat Gaziosmanpaşa University, Tokat 60250, Turkey; 2 Department of Chemistry, Faculty of Science and Letters, Tokat Gaziosmanpaşa University, Tokat 60250, Turkey; 3 Bioengineering Division, Institute of Graduate Studies, Tokat Gaziosmanpaşa University, Tokat 60250, Turkey

**Keywords:** denture base resin, polymethyl methacrylate, glass fiber mesh, polypropylene hernia mesh, aluminum mesh, reinforcement

## Abstract

The aim of this study was to investigate the potential use of polypropylene (PP) hernia mesh as a reinforcement of PMMA denture base resin in comparison with metal and glass fiber meshes, with the expectation of enhancing the mechanical stability of the PMMA dentures in oral conditions. The control group with no mesh, the aluminum metal mesh (Al) group, the PP1 (PP mesh used on top) group, the PP2 (PP mesh used on both the top and bottom) group, the orthopedic casting tape with self-curing resin (DP0) group, and the flushed form (DPA) group were fabricated in a chromium mold. A total of 144 specimens were divided into three equal portions and subjected to: first, no treatment; second, thermal cycling only; and third, thermal cycling and chewing simulation. The flexural strength, maximum deformation, and flexural modulus were determined by a three-point bending test to compare mechanical properties. Fracture surfaces were evaluated by scanning electron microscopy. The obtained data were statistically analyzed by a two-way ANOVA test with Bonferroni corrections. The non-treated Al mesh reinforcement group exhibited the highest (82.66 ± 6.65 MPa) flexural strength, and the PP2 group treated with chewing simulation displayed the lowest (56.64 ± 4.59 MPa) flexural strength. The Al group showed the highest (7.25 ± 1.05 mm) maximum deformation and the PP2 group showed lowest (3.64 ± 0.28 mm) maximum deformation when both groups were not subjected to any treatment. The control group with no treatment exhibited the lowest (1556.98 ± 270.62 MPa) flexural modulus values, and the Al group with no treatment exhibited the highest (3106.07 ± 588.68 MPa) flexural modulus values. All the mesh groups displayed intact fractures. Any type of mesh used for reinforcement exhibited a significant change in all flexural properties (*p* < 0.001). The PP1 reinforcement group did not exhibit a significant change in mechanical properties when the effect of treatment was compared. Using PP hernia mesh on top enhanced the mechanical properties despite the weakening when it was used on both the top and bottom. The mechanical stability provided by the PP hernia mesh indicated it to be a promising candidate to be used for reinforcement.

## 1. Introduction

Heat-polymerized polymethyl methacrylate (PMMA) has been the most widely used denture base resin in prosthetic dentistry since its introduction in the early 1940s due to its positive properties such as biocompatibility, dimensional stability, non-toxicity, tastelessness and odorlessness, stability in the oral environment, aesthetic appearance, resistance to color change, ease of processing, and low cost [1,2,3,4,5,6]. Despite its unique features, it has undesirable properties such as weakness in bending strength and impact resistance, fatigue fracture as a result of the cyclic chewing forces it is exposed to, and breakage of the prosthesis because of accidental falling on rigid surfaces [7,8,9,10]. It has been reported that two-thirds of the denture bases were broken, mainly due to impact failure a few years after they were made [11].

Enhancing the physical properties of the polymers is an important challenge, and the preparation of composites of polymers with particles and fibers is a widely studied strategy [12,13,14,15,16,17]. Various strategies were performed in order to enhance the mechanical properties of PMMA dentures by using them as composites of PMMA with various micro- nano-particles, fibers or wires, and meshes [8,10,18,19,20,21,22]. An alternative to these strategies was the modification of the chemistry of PMMA by graft copolymerization with styrene butadiene, which exhibited a lower bending strength, unlike the higher impact resistance, compared to conventional PMMA resin [23,24]. The main problem of using particulate fillers, fibers, and wires was the poor adhesion between the PMMA matrix and the surfaces of these particulates, fibers, and wires [8,10,18,19,20]. Using types of fibers such as aramid carbon or graphite fibers leads to poor aesthetics and weak adhesion to the PMMA matrix [25].

Metal (stainless steel, cobalt-chromium alloy) meshes were used as an enhancer of mechanical properties in several studies [18,26,27]. Metal mesh usage did not receive widespread acceptance by dentists due to recurring fractures [28]. Alternatively, glass and polymer (high-performance polymer-BioHPP, nylon) fiber mesh were used for denture base reinforcement [25,27,28,29,30]. Rana et al. [25] reported using orthopedic casting tape made of a glass fiber framework as an alternative mesh product for the reinforcement of denture base resin. However, aesthetic concerns may limit the use of these metal, glass, and polymer meshes as reinforcement, since appearance may differ according to the thickness of the mesh used compared to pure resin.

Polypropylene (PP) is a biocompatible polymer and is used in many biomedical products because of its favorable properties such as inertness, elasticity and tensile strength, durability in acid and alkali media, low density (0.91 g/cm^3^), and low cost, which make it a suitable candidate to be used for PMMA base resin reinforcement [31,32]. PP mesh is one of these products; it is a surgical mesh commonly used to repair different types of hernia [33].

This study aimed to investigate the potential use of PP hernia mesh as a reinforcement of PMMA denture base resin in comparison with metal and glass fiber meshes that were reported in the literature to be used for the reinforcement of heat-polymerized PMMA denture base resin. The mesh materials, especially PP, were not further surface-treated to enhance the adhesion of PMMA resin to these fiber meshes, because the treatment chemistry is another variable that is strongly dependent on many parameters.

## 2. Materials and Methods

Heat-cured PMMA denture base resin (Meliodent, Kulzer GmbH, Frankfurt, Germany) was used. PP mesh (4A Medikal, Ankara, Turkey) was obtained in a sterile package that is produced for surgical operations. Aluminum (Al) mesh (Polywax, Bilkim Chemical Company, İzmir, Turkey) was used as received. Delta Plus orthopedic cast (Delta Lite Plus, BSN Medical GmbH, Frankfurt, Germany) was obtained in a sealed package and used in two groups: first, without any treatment after curing; and second, washed with ethanol to get rid of the curing resin initiated by air contact. The cleansing with the alcohol procedure is as follows: the material was cut in dimensions suitable to fit in the two-piece mold (in 100 × 210 × 33 mm^3^ dimensions with a 65 × 150 × 3 mm^3^ sample space), immersed in an excess amount of ethanol (96% *v/v*), swung with gentle movements periodically within the first two hours and kept in for 24 h, washed with fresh ethanol, and dried prior to use. The physical properties of the materials used are summarized in Table 1.

Five mesh-reinforced groups and a control group with no reinforcement were formed, and each group was divided into three subgroups that received three treatments: (i) no treatment, (ii) thermal cycling, and (iii) thermal cycling and chewing simulation. They were applied on the test specimens of these six groups. The mesh-reinforced groups were: the aluminum mesh group (Al), the single-layer PP group (PP1), the two-PP-layers group (one on the top and one on the bottom of the specimen) (PP2), the Delta Plus glass fiber mesh without cleansing group (DP0), and the Delta Plus glass fiber mesh washed with ethanol group (DPA).

The ideal powder-liquid mixing ratio was used (25 g powder and 10 mL liquid), and complete wetting was observed. The acrylic paste was cast using a mold made of chrome designed with the dimensions of 65 × 150 × 3 mm^3^. Then, the mold was pressed in the hydraulic press device (GLS, Gulersan Lubrication Equipment Industry and Trade Co. Ltd., Istanbul, Turkey) for 5 min, and the excess acrylic was removed. Then, the polymerization was completed by making temperature and time adjustments in accordance with the manufacturer’s instructions. Three-point bending test specimens were cut in 65 × 10 × 3 mm^3^ dimensions using a laser cutting machine (LazerFix Laser Technology, Konya, Turkey) in order to match the dimensions described in the ISO 178 standard [34]. All specimens were stored in distilled water for 24 h. In this way, residual monomers were removed.

The thermal cycling group specimens were aged in distilled water baths of 5 °C and 55 °C with a dwell time of 30 s for 10,000 cycles in a thermocycler machine (Thermocycler THE1100, SD Mechatronic GmbH, Feldkirchen-Westerham, Germany), which is equivalent to the use of dentures in the mouth for 1 year [35].

A chewing simulation was performed on specimens subjected to thermal cycles using a chewing simulator device (Chewing Simulator CS-4.2, SD Mechatronic GmbH, Munich, Germany). Chewing simulation was carried out for 240,000 cycles, which is equivalent to the use of dentures in the mouth for 1 year [36]. Chewing forces were applied under distilled water to prevent overheating, and standard metal spheres were used as the abrasive tip. Each sample was loaded at a frequency of 1.2 Hz with vertical and lateral movements of 2 mm. The vertical load value was maintained at 5 kg during the motion, which is equivalent to 49 N of effective masticating force, which is used as a standard [37,38].

**Figure 1:**
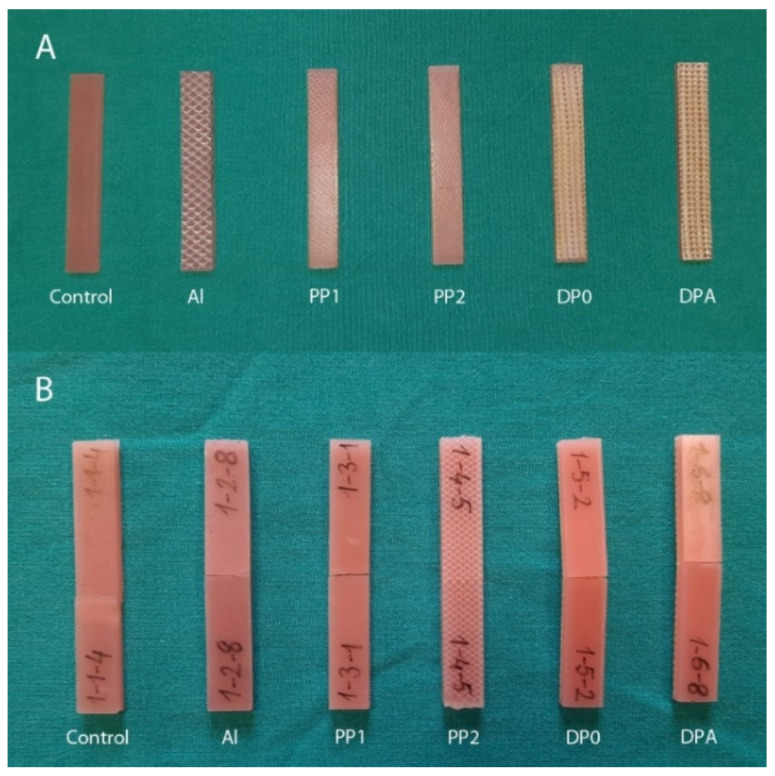
Test specimens before (**A**) and after the three-point bending test (**B**).

The flexural properties of the control and mesh-reinforced groups were determined by a three-point bending test in accordance with the ISO 178 standard. A representative image of the test specimens before and after the three-point bending test is shown in Figure 1. The three-point bending tests were performed on a total of 144 samples in 18 groups, with *n* = 8 samples for each group, using a universal testing machine (Autograph AGS-X, Shimadzu, Japan) at a compression rate of 3 mm/min. The samples placed between the shoulders had a gap of 50 mm. All tests were carried out at room temperature.

The morphologies of the fracture surfaces were investigated using scanning electron microscopy (SEM) images taken from the fracture surfaces of the specimens, which were obtained after the three-point bending tests. The SEM samples were prepared by cutting the fractured test specimens to reduce the height to 5 mm long. The SEM samples were positioned on the sample tray by facing the fracture surfaces upward and then coated with gold (Au) under a vacuum atmosphere using a coating instrument (Quorum Q150R ES, Birmingham, UK). The SEM images were collected using SEM equipment (Tescan Mira3 XMU, Brno, Czechia) with an accelerating voltage of 10 kV.

All the statistical analyses were carried out using statistics software SPSS 20 (IBM, Chicago, IL, USA). The flexural strengths obtained by the three-point bending tests for all groups were analyzed using the two-way ANOVA test, and the level of significance was set to *p* < 0.05. Bonferroni corrections were performed for multiple comparisons.

## 3. Results

### 3.1. Mechanical Tests

**Figure 2:**
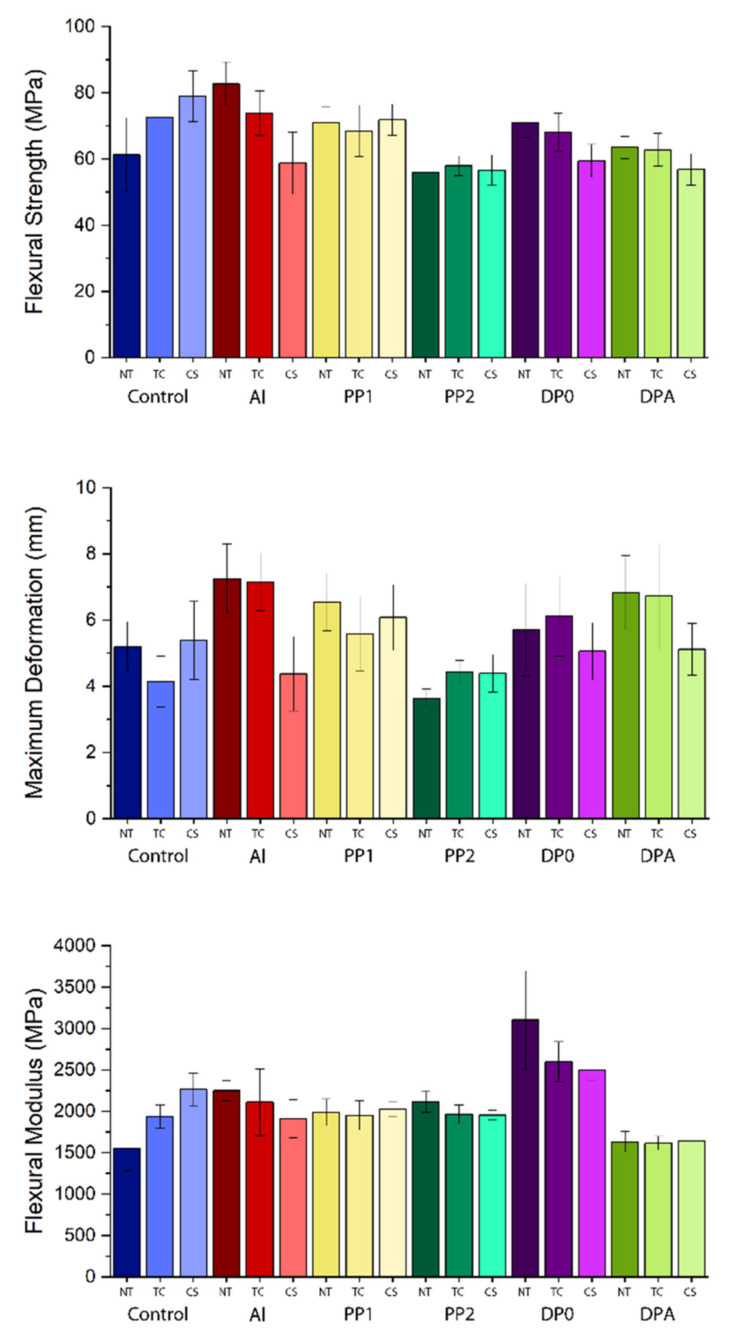
Test specimens before and after the three-point bending test.

The test results for the between-subject effects obtained by the two-way ANOVA analysis of the three-point bending test results for three flexural properties—strength (MPa), maximum deformation (mm), and modulus (MPa)—are summarized in Table 2. The means with standard deviations and scoring on the significance of the tested variables obtained by comparisons of the three-point bending test results are presented in Table 3 (in group comparison; treatment regardless of mesh reinforcement or mesh reinforcement regardless of treatment) and Table 4 (pairwise comparisons). The test results obtained by the three-point testing were plotted and are presented in Figure 2. According to the test results, any type of mesh used for reinforcement exhibited a significant change in all flexural properties of the denture base resin (*p* < 0.001). Treatments on the PMMA denture base resins resulted in a significant change in flexural strength and maximum deformation (*p* < 0.05) and no significant difference in flexural modulus (*p* > 0.05).

By comparing the treatments regardless of reinforcement, the chewing simulation samples exhibited a significant decrease in flexural strength and maximum deformation (*p* < 0.05). PP2 and DPA exhibited a significant decrease in flexural strength compared to the other groups regardless of the treatment applied (*p* < 0.05). Maximum deformation significantly increased for the Al, PP1, and DPA groups (*p* < 0.001). DPA exhibited a significant decrease and DP0 exhibited a significant increase in flexural modulus regardless of the treatment (*p* < 0.001).

The pairwise comparisons revealed that the thermal treatment and chewing simulation caused the flexural strength of the control specimens to increase significantly compared to the non-treated ones. Conversely, each treatment on the Al groups exhibited a significant decrease in flexural strength (*p* < 0.05). The treatments did not exhibit any significance for the remaining mesh reinforcement groups, except for chewing simulation on the DP0 group, in which the flexural modulus decreased (*p* < 0.05).

The greatest value of flexural strength was observed as 82.66 ± 6.65 MPa for the non-treated Al mesh reinforcement group (Figure 2).

The non-treated Al, PP1, and DP0 mesh reinforcement groups showed significantly better flexural strengths compared to the control (*p* < 0.05). Thermal-cycled PP2 exhibited a lower flexural strength than the control, Al, PP1, and DP0 mesh reinforcement groups (*p* < 0.05). Chewing simulation resulted in a decrease in flexural strength for all groups except for the control and PP1 groups (*p* < 0.001). The change in maximum deformation remained insignificant, except for chewing simulation on the Al and DPA groups, which was observed as reducing deformation when compared to treatments for each mesh type. By comparing the maximum deformation for different meshes within the non-treated group, the maximum deformation significantly decreased for the PP2 group and significantly increased for the Al and DP0 groups (*p* < 0.05). The test results of the thermal-cycled specimens of all mesh groups besides PP2 exhibited a significant difference compared to the control group (*p* < 0.05). The greatest maximum deformation value for the thermal-cycled test group was observed for the Al mesh-reinforced group: 7.16 ± 0.87 mm. The maximum deformation of the PP1 group, which has the highest maximum deformation (6.08 ± 0.99 mm) among chewing simulation specimens, exhibited a significant difference compared to the Al and PP2 groups, which had lower maximum deformation values (*p* < 0.05). The effect of the treatment on the flexural modulus of the control group demonstrated a significant increase for both thermal cycling (*p* < 0.05) and chewing simulation (*p* < 0.001) compared to no-treatment. Chewing simulation on the Al mesh group resulted in a significant decrease in the flexural modulus compared to the non-treated Al mesh group, from 2251.24 ± 124.29 MPa to 1912.68 ± 228.39 MPa (*p* < 0.05). Thermal cycling on the DP0 group caused a decrease in the flexural modulus from 3106.07 ± 588.68 MPa to 2598.06 ± 243.44 MPa (*p* < 0.001); however, chewing simulation did not exhibit significance compared to thermal-cycled DP0 group (*p* > 0.05). The treatments did not change the flexural moduli of both the PP1 and DPA groups at a level of significance. By comparing the non-treated test groups with each other, all mesh groups except for DP0 enhanced the flexural modulus of the PMMA denture bas resin (*p* < 0.05). The DP0 and DPA specimens of thermal cycling exhibited a significant difference in the flexural modulus compared to the other test groups, namely, it was greater for DP0 (*p* < 0.001) and lower for DPA (*p* < 0.05). The flexural modulus of the DP0 (*p* < 0.001) and control groups was observed to be significantly greater than that of the other mesh reinforcement groups, except for the control group.

### 3.2. SEM Analysis

**Figure 3:**
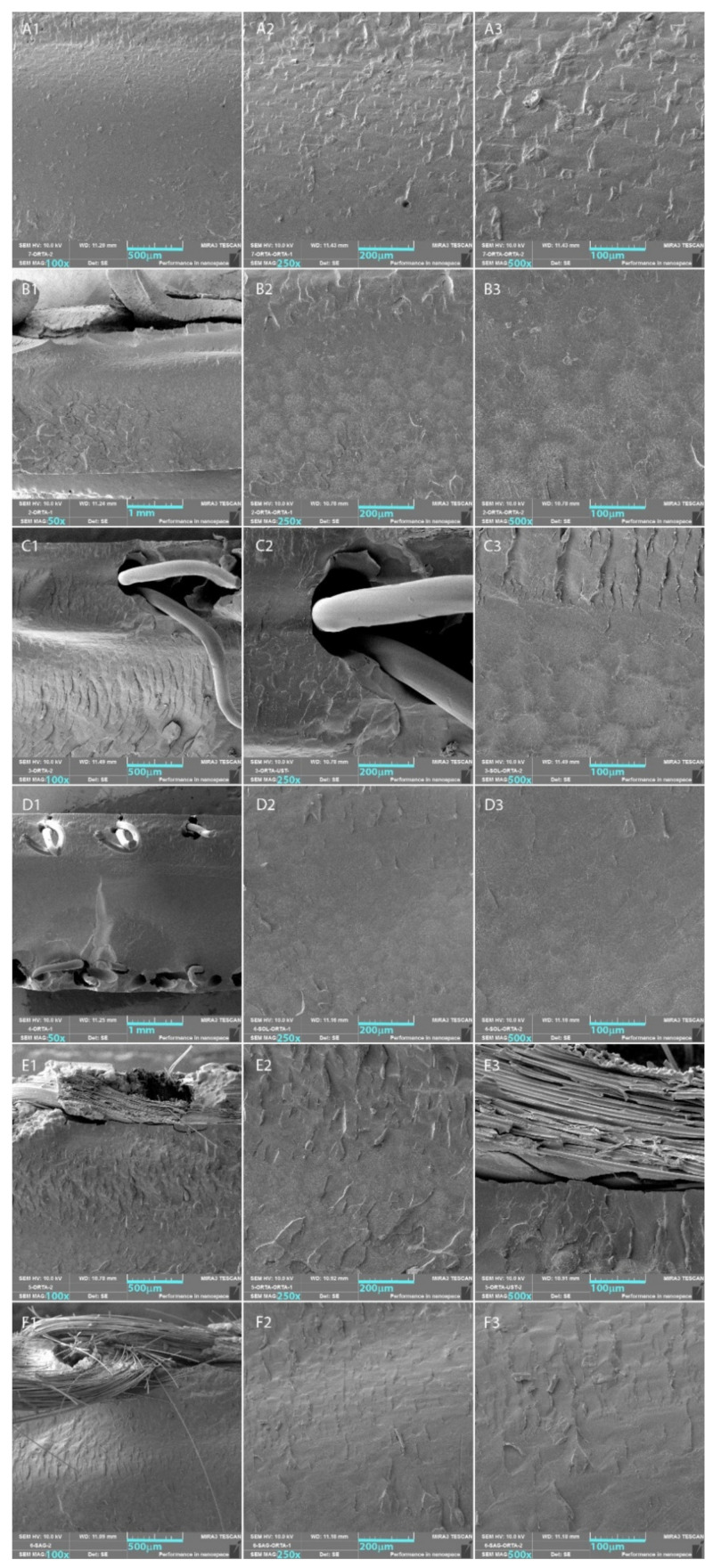
SEM images of the test groups—(**A**) Control, (**B**) Al, (**C**) PP1, (**D**) PP2, (**E**) DP0, (**F**) DPA—with different magnifications: (1) 100× or 50×, (2) 250×, (3) 500×.

SEM images with different magnifications were collected from the surfaces of the fractures, which belong to the three-point bending test specimens for the control and mesh reinforcement groups. The SEM images of fracture surfaces with three different magnifications (100×, 250×, and 500×) for each test group are presented in Figure 3.

It was observed that a brittle fracture was evident from the SEM images of the control group. River line patterns were aligned to the point of the force applied and were almost perpendicular to the surface. These patterns were in the range of 10–30 µm long and were distributed along the material that resulted in a rough fracture surface. On the crack surface of the metal-reinforced PMMA denture base resin, a more complex pattern was observed. A river line-like pattern was evident in the close neighborhood of the metal mesh, and heterogeneously sized fisheye patterns were observed along the counter distance of the metal mesh. The SEM images obtained from the PP1 group showed a similar behavior to that of the Al group, except the depth and length of the river patterns were greater. Two layers of PP mesh within both opposite surfaces were evident in the SEM images of the PP2 mesh group. Although a similar behavior was observed on the fracture surface, the only difference compared to the Al and PP1 groups was that the concentration of the patterns was lower. The SEM images of the DP0 group clearly indicate that the self-curing resin on the glass fibers reside in the close neighborhood of the glass fiber clusters, and it provided a hard cast structure to the glass fiber mesh. It was also observed that the interface between the PMMA matrix and the cured polymer surrounding the glass fiber mesh had detached during the three-point bending test. River patterns, which are characteristic of a brittle fracture mechanism, were observed below the mesh structure along the PMMA denture base resin. The SEM images of the DPA group confirmed the successful leaching of the self-curing resin prior to use as reinforcement. The glass fiber bundles apparently separated in the DPA specimen despite the DP0 specimens, in which the glass fiber bundles packed closely within the self-cured resin.

## 4. Discussion

In this study, three different mesh reinforcements were set in five groups: one metal mesh group, two PP mesh groups based on mesh location, and two woven glass fiber mesh groups based on casting self-curing resin and its flushed form. They were used with heat-polymerized PMMA denture base resin. A control group with no mesh was set for the conduction of comparisons with each mesh-reinforced group. Using mesh structures to enhance the mechanical properties of the PMMA denture base resins became preferable due to the ease of placing the fibers precisely compared to the difficulty of positioning the fibers in parallel orientation and preventing them from dispersing during the pack and press process [30,39]. Three mechanical properties, namely, flexural strength, maximum deformation, and flexural modulus, were obtained by three-point bending tests, and these were compared with each other. It should be noted that all of the test specimens prepared with mesh embedding during the pack and press process exhibited fractures with intact pieces. We decided to use the test specimens with 65 × 150 × 3 mm^3^ dimensions instead of using test specimens obtained by the 3D model of the high or low alveolar ridge. The reason for this preference is that the forces causing fracture are highly dependent on the thickness and irregularities of the specimens, even those produced with the same 3D model. Therefore, comparing the flexural strength and other physical properties obtained by the test specimens (the dimensions of each had been input during three-point bending test) would be more consistent. All of the test specimens were kept in distilled water for two months after fabrication and prior to testing according to the storage periods reported [5,30,40,41,42,43,44]. It was reported that the reduction in flexural strength occurs in the first four weeks of immersion [45,46].

The most commonly used form of denture base reinforcement is made using metal mesh structures. Despite its capacity to provide higher flexural strengths, concerns arise regarding aesthetic issues because of the dark shadowed color seen due to the metal mesh used [47,48] and the inadequate adhesion of acrylic resin on the metal surface [23]. It has been reported that many reinforced prostheses resulted in fracture because of the poor adhesion between the denture base and the reinforcement material, regardless of the reinforcement material and the denture base [29,44,49,50]. Using Al, single-layer PP, and DP0 enhanced the flexural strength of the non-treated specimens. The flexural strength together with the flexural modulus of the control specimens increased significantly by both thermal cycling and chewing simulation treatments, but there was no significant change observed in its maximum deformation. Al mesh reinforcement provided the greatest flexural strength (82.66 ± 6.65 MPa) to the heat-cured PPMA denture base resin, which is similar to the results reported by Balch et al. [48], and this strength was reduced by both the thermal cycling (73.87 ± 6.73 MPa) and chewing simulation (58.76 ± 9.34 MPa) treatments. The poor adhesion of the acrylic polymer on the metal surface was further reduced by the vibrations of the microstructure exposed to cyclic temperature changes and cyclic forces. However, thermal cycling did not change the maximum deformation, but chewing simulation resulted in a significant decrease (4.38 ± 1.12 mm), and the PMMA resin became brittle. Similarly, the flexural modulus of the Al mesh group decreased significantly by chewing simulation (1912.68 ± 228.39 MPa), which can be explained by the reduced adhesion between the Al mesh and the PMMA matrix due to vibrations across the microstructure because of the cyclic forces applied during these treatments.

The rationale for using the PP fiber mesh structure as denture base resin reinforcement was its unique properties such as its very light weight, high strength and modulus, resistance to chemical deterioration, resistance to abrasion, resistance to the absorption of moisture, resilience, and non-brittle character [10,51]. The treatments on the PP mesh-embedded specimens of the PP1 and PP2 groups did not exhibit a significant change in all three mechanical properties compared with the thermal cycling and chewing simulation groups. Non-treated PP1 exhibited a significant increase in flexural strength (71.01 ± 4.70 MPa) compared to the control (61.28 ± 11.17 MPa). The maximum deformation values observed for all treatments were significantly different for the PP1 group compared to the PP2 group, which caused the PP1 group to exhibit a relatively ductile character. The PP meshes used on both sides of the PP2 group specimens resulted in a less ductile character and a brittle fracture due to the localization of stress around the mesh located at the surface opposite to the surface where the compressive force was applied. Using PP meshes on both the top and bottom surfaces of the PP2 samples does not seem to be applicable due to the less ductile character provided by the PMMA resin.

It has been reported by many authors that using glass fibers or their woven mesh forms enhances the mechanical strength of denture base resins [1,29,41,42,43,52]. The orientation, position, volume, stress, and degree of resin impregnation are important parameters influencing the mechanical strength of the denture base resin [53,54]. The use of orthopedic casting tape as reinforcement of the acrylic denture base resins was reported to be effective in strengthening the heat-polymerized PMMA denture base resin [25]. It is known that the orthopedic casting tapes consist of woven multifilament glass fiber mesh surrounded by self-curing polymer resin that is cured after initiation by air contact. In this present study, two groups (DP0 and DPA) were formed to investigate the effect of this self-curing resin on the mechanical properties provided to the PMMA denture base resin. DP0 exhibited a significant increase in flexural strength (71.04 ± 4.62 MPa) and flexural modulus (3106.07 ± 588.68 MPa), which was similarly previously reported by Rana et al. [25], both of which were significantly decreased by both thermal cycling and chewing simulation. This behavior of the DP0 group can be attributed to the compatibility and/or high mechanical strength of the self-curing polymer surmounting the glass fibers compared to its flushed form DPA, which did not exhibit a significant change in the mechanical properties. The DPA groups did not exhibit a significant difference in flexural properties, except for the flexural modulus. The flexural moduli of the DPA specimens were significantly lower than those of the DP0 group for all three treatment groups. As can clearly be seen in the SEM images of the DP0 and DPA groups, the self-curing resin was evident around the woven glass fiber network for the DP0 group. The greater values of the flexural modulus of the DP0 groups compared to those of the DPA groups may be associated with the effective integration of the self-curing resin surrounding the glass fiber mesh with the heat-polymerized PMMA denture base resin.

By comparing the aesthetic view of the mesh-embedded PMMA denture base resin samples (Figure 1), it can be seen that using Al and glass fiber mesh as reinforcement may not be favorable due to the unnatural texture and color view on the surface. The thin fibers forming the PP mesh provided a more favorable aesthetic view compared to the others.

## 5. Conclusions

In this study, we aimed to investigate the potential use of PP hernia mesh structures (used on the top layer only and on both the top and bottom layers) as a reinforcement of heat-polymerized PMMA denture base resin and compared it to resins with no reinforcement and Al mesh-reinforced and orthopedic cast tape (made of glass fiber mesh)-reinforced resin. The Al mesh-reinforced group displayed the highest flexural strength values, and PP2 group treated with both thermal cycling and chewing simulation displayed the lowest flexural strength values. The most ductile samples were in the Al group, and least ductile samples were in the PP2 reinforcement group, both of which were untreated. The DP0 group displayed the highest flexural modulus, and the control group displayed the lowest flexural modulus, and both groups were untreated. Flushing the self-curing resin surmounting the glass fibers in DPA caused the mesh to exhibit a significant decrease in the flexural modulus for all treatment groups. Thermal cycling and thermal cycling followed by chewing simulation did not exhibit a significant change in all three properties for PP1, which can be said to keep its mechanical stability under these stress conditions. The PP hernia mesh can be considered as a candidate for reinforcement material because of this behavior and the aesthetic view of the PP mesh-reinforced PMMA denture base resin. Additionally, this reinforcement can further be enhanced by surface treatment and/or the grafting of functional monomers on PP mesh, which may lead to the obtention of a PP mesh cross-linkable with PMMA.

## Figures and Tables

**Table 1:** Physical properties of the Al, PP, and GF materials.

Material	Density g/cm^3^	Tensile Strength (MPa)	Elongation at Break (%)
Al	2.7	54.62	32.54
PP	0.9	14.24	53.13
DPA	2.5	34.87	36.68

**Table 2:** Test results for between-subject effects obtained by two-way ANOVA analysis.

Variable	Comparison	Sum of Squares	df	Mean Square	F	*p*
Flexural strength	Treatment	432.343	2	216.171	5.548	0.005 *
	Mesh reinforcement	4438.059	5	887.612	22.780	0.000 *
	Treatment x mesh reinforcement	4047.818	10	404.782	10.389	0.000 *
Maximum deformation	Treatment	16.831	2	8.416	8.537	0.000 *
	Mesh reinforcement	86.273	5	17.255	17.503	0.000 *
	Treatment x mesh reinforcement	59.213	10	5.921	6.007	0.000 *
Flexural modulus	Treatment	161,098.685	2	80,549.342	1.565	0.213
	Mesh reinforcement	15,999,143.508	5	3,199,828.702	62.182	0.000 *
	Treatment x mesh reinforcement	4,155,670.119	10	415,567.012	8.076	0.000 *

*: Statistically significant difference.

**Table 3:** Mean, standard deviations, and significance of three mechanical properties by comparisons of the three-point bending test results.

Treatment or Mesh Group	Flexural Strength (MPa)	Maximum Deformation (mm)	Flexural Modulus (MPa)
No treatment	67.58 ± 10.47 ^(x)^	5.86 ± 1.53 ^(x)^	2109.42 ± 582.13 ^(x)^
Thermal cycling	67.33 ± 8.08 ^(x)^	5.70 ± 1.50 ^(x)^	2030.30 ± 362.65 ^(x)^
Chewing simulation	63.78 ± 10.49 ^(y)^	5.07 ± 1.07 ^(y)^	2051.45 ± 310.46 ^(x)^
Control	70.96 ± 11.43 ^(a)^	4.91 ± 1.04 ^(de)^	1919.50 ± 356.70 ^(b)^
Al	71.76 ± 12.47 ^(a)^	6.26 ± 1.67 ^(ab)^	2091.22 ± 300.41 ^(b)^
PP1	70.46 ± 5.82 ^(a)^	6.08 ± 1.04 ^(ac)^	1991.47 ± 143.56 ^(b)^
PP2	56.88 ± 3.46 ^(b)^	4.16 ± 55 ^(e)^	2013.36 ± 125.12 ^(b)^
DP0	66.24 ± 7.02 ^(a)^	5.63 ± 1.21 ^(bcd)^	2735.07 ± 449.88 ^(a)^
DPA	61.07 ± 5.19 ^(b)^	6.23 ± 1.41 ^(a)^	1631.73 ± 115.94 ^(c)^

Comparisons—(xyz): for treatment; (abcde): for mesh reinforcement groups. Values with different superscript letters indicate significant differences.

**Table 4:** Mean, standard deviations, and significance by pairwise comparisons of the three-point bending test results.

Treatment	Mesh Group	Flexural Strength (MPa)	Maximum Deformation (mm)	Flexural Modulus (MPa)
No treatment	Control	61.28 ± 11.17 ^(y,c)^	5.20 ± 0.75 ^(x,b)^	1556.98 ± 270.62 ^(z,c)^
	Al	82.66 ± 6.65 ^(x,a)^	7.25 ± 1.05 ^(x,a)^	2251.24 ± 124.29 ^(x,b)^
	PP1	71.01 ± 4.70 ^(x,b)^	6.55 ± 0.87 ^(x,ab)^	1991.45 ± 160.91 ^(x,b)^
	PP2	55.98 ± 2.66 ^(x,c)^	3.64 ± 0.28 ^(x,c)^	2117.56 ± 130.41 ^(x,b)^
	DP0	71.04 ± 4.62 ^(x,b)^	5.70 ± 1.40 ^(x,ab)^	3106.07 ± 588.68 ^(x,a)^
	DPA	63.49 ± 3.38 ^(x,bc)^	6.84 ± 1.11 ^(x,a)^	1633.25 ± 122.86 ^(x,c)^
Thermal cycle	Control	72.60 ± 7.84 ^(x,a)^	4.15 ± 0.77 ^(x,c)^	1936.88 ± 138.48 ^(y,b)^
	Al	73.87 ± 6.73 ^(y,a)^	7.16 ± 0.87 ^(x,a)^	2109.74 ± 403.53 ^(xy,b)^
	PP1	68.49 ± 7.75 ^(x,ab)^	5.59 ± 1.13 ^(x,b)^	1952.45 ± 174.58 ^(x,b)^
	PP2	58.03 ± 2.96 ^(x,c)^	4.44 ± 0.34 ^(x,c)^	1966.98 ± 110.54 ^(x,b)^
	DP0	68.16 ± 5.79 ^(x,ab)^	6.12 ± 1.20 ^(x,ab)^	2598.06 ± 243.44 ^(y,a)^
	DPA	62.82 ± 4.94 ^(x,bc)^	6.72 ± 1.60 ^(x,ab)^	1617.71 ± 86.06 ^(x,c)^
Chewing simulation	Control	78.99 ± 7.70 ^(x,a)^	5.39 ± 1.18 ^(x,ab)^	2264.64 ± 197.42 ^(x,a)^
	Al	58.76 ± 9.34 ^(z,b)^	4.38 ± 1.12 ^(y,b)^	1912.68 ± 228.39 ^(y,bc)^
	PP1	71.89 ± 4.71 ^(x,a)^	6.08 ± 0.99 ^(x,a)^	2030.53 ± 88.67 ^(x,b)^
	PP2	56.64 ± 4.59 ^(x,b)^	4.39 ± 0.57 ^(x,b)^	1955.54 ± 59.41 ^(x,bc)^
	DP0	59.51 ± 4.98 ^(y,b)^	5.06 ± 0.86 ^(x,ab)^	2501.07 ± 133.68 ^(y,a)^
	DPA	56.90 ± 4.74 ^(x,b)^	5.12 ± 0.78 ^(y,ab)^	1644.24 ± 145.81^(x,c)^

Comparisons—(xyz): for treatments and compared with the same mesh with the same background color; (abc): for mesh reinforcement groups compared with different meshes in the same treatment groups. Values with different superscript letters indicate significant differences.

## Data Availability

Not applicable.
